# Supplementary material for: Learning curve and surgical outcome of robotic assisted colorectal surgery with ERAS program
Source: Sci Rep. 2022 Nov 29;12:20566. doi: 10.1038/s41598-022-24665-w (PMC9709162; doi:10.1038/s41598-022-24665-w)
Supplement: Supplementary file 2 — Supplementary Information 2. [file 41598_2022_24665_MOESM2_ESM.zip › RAL Raw data-Ñ[▒K/109/OP N_ñw╝╨░O▒Kñσ.pdf]

W52 / W52

臺中榮民總醫院
手術室護理記錄

[redacted]

基本資料

- 室別： 開刀房-17室，手術科別： CRS，手術類別： 常規，日期： 2021/08/26
- 進入時間： 0725 ； 麻醉開始時間： 0740 ； Time Out時間： 0855 ； 劃刀時間： 0856 ； 手術結束時間： 1250 送出時間： 1255 ；
- 麻醉方式： GE
  診斷：Colorectal cancer with distance metastasis
  手術名稱：Robot Assisted Colectomy(L)
  手術局部用藥：2%Xylocaine jelly
  術前預防性抗生素：
  - 抗生素名稱一： Veterin 1GM IVD/由麻醉護理人員給予
  - 時間： 0845
  - 上次簽章: 吳婉寧

  ，追加預防性抗生素：
- 手術醫師： 蔣鋒帆，楊曙榮，洪浚嚴，林志安
- 麻醉醫師： VS 陳永銘
-
  - 刷手護士A ： 王明盈，時間： 0720，刷手護士B ： 歐真勤，時間： 0720
  - 流動護士A ： 吳婉寧，時間： 0720，接替流動一： 歐真勤，時間： 1130

皮膚狀況

A-V Shunt： 無
術前： 完整
記錄者： 上次簽章: 吳婉寧
術後： 完整
記錄者： 上次簽章: 歐真勤

手術準備

手術臥式 單一手術臥式

-
  - 手術臥式： 截石臥式
  - 使用附件： 溫毯 0 ℃，矽膠枕頭，腳架
  - 皮膚準備： Hibiscrub，2% Chlorhexidine
  - 電燒： 使用電燒: 電極片位置： 其它： 迴路電極墊，雙極電燒，記錄者： 上次簽章: 吳婉寧

止血帶T： 無

植入物

無

標本送檢

- 細菌培養 無
- 細胞學 無
- 冰凍切片 無
- 病理標本
  組織名稱一：Distal cut end
  ,
  組織名稱二：s-colon
  ,
  組織名稱三：IMA root
- 截肢 無

標本交付 標本： 1 袋，標本： 0 盒，標本： 2 瓶，細胞學： 0 管，Culture Tube： 0 管，被交付醫師： R 林志安，

- 記錄者： 上次簽章: 歐真勤

- 時間： 1150

傷口術後敷料

傷口敷料：

傷口敷料： 無

- 請於圖上標示部位：

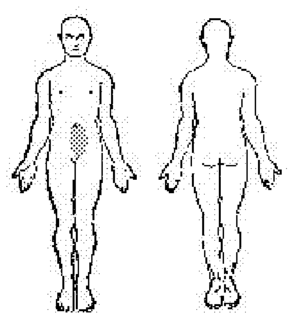

腹部mepore洞

- 傷口敷料： mepore

導管

導管	天數	導管資訊	插入日期	插入時間	更換日期	拔除日期	拔除時間	拔除原因	預計拔除/更換	預計拔除/更換日期
Sump NG Tube(1)	1	號碼:16 Fr.; 深度:Fix 60cm; 部位:胃;	2021/08/26	0830						
A-line (1)	1	號碼:20 G; 部位:左手;	2021/08/26	0815						
Foley(1)	1	號碼:16 Fr.; 部位:膀胱;	2021/08/26	0820						
Double Lumen CVC(1)	1	號碼:7fr; 深度:15cm; 部位:jugular (右); 象限:RU;	2021/08/26	0830						

用物計數

- 手術中出血量： minimal c.c.
- 手術中尿量： 100 c.c.
-
  - 其它： 無

用物計數： 多切口同時縫合

- 1.器械： 正確，2.縫針： 正確，3.刀片： 正確，4.紗布： 正確，5.腹部墊： 未使用，6.紗球： 未使用，7.PEANUTS ： 未使用，8.紗條： 未使用，9.絲帶： 未使用，10.針頭： 未使用，11.Vessel loop： 未使用，12.Boots： 未使用，13.Jelco： 未使用，14.棉條： 未使用，
  - 第一次：
    - 刷手護士 ： 王明盈
    - 流動護士： 上次簽章: 歐真勤
    - 時間： 1130

  ,
  - 第二次：
    - 刷手護士 ： 王明盈
    - 流動護士： 上次簽章: 歐真勤
    - 時間： 1140

  ,
  - 第三次：
    - 刷手護士 ： 王明盈
    - 流動護士： 上次簽章: 歐真勤
    - 時間： 1150

備註

備註：XI達文西30度鏡:1號

- 記錄者： 上次簽章: 吳婉寧

- 時間： 0920

填寫護理人員_歐真勤

正式摘要

	W52 / W52

doctorRank:B
